# Longitudinal modeling of health-related quality of life trajectories over 12 months following road trauma

**DOI:** 10.1371/journal.pone.0336144

**Published:** 2025-11-21

**Authors:** Somayeh Momenyan, Herbert Chan, Shannon Erdelyi, Lulu X. Pei, Lina Jae, John A. Taylor, John A. Staples, Jeffrey R. Brubacher

**Affiliations:** 1 Department of Emergency Medicine, Faculty of Medicine, University of British Columbia, Vancouver, British Columbia, Canada; 2 Department of Medicine, University of British Columbia, Faculty of Medicine, Vancouver, British Columbia, Canada; 3 Centre for Clinical Epidemiology & Evaluation, Vancouver, British Columbia, Canada; Jordan University of Science and Technology, JORDAN

## Abstract

**Introduction:**

This study identified the potential phases of health-related quality of life (HRQoL) change over a year following road trauma (RT) injury, as well as its predictors.

**Methods:**

This inception cohort study recruited 1480 Canadian RT survivors from July 2018 to March 2020. HRQoL outcome was assessed with the 5-level version of the EuroQol (EQ-5D-5L) instrument at baseline (pre-injury) and at 2, 4, 6, and 12 months post-injury. Predictors of HRQoL included sociodemographic, psychological, medical, and trauma-related factors collected at baseline. We applied generalized additive mixed models to flexibly capture nonlinear changes in HRQoL over time, and piecewise latent growth curve model to analyze distinct linear phases of recovery across defined time intervals.

**Results:**

The estimated trajectory of EQ-5D-5L summary and EQ-VAS scores were lower than baseline at 2-months (phase 1), and then increased (phase 2), but did not return to baseline values at 12-months. White ethnicity, higher somatic symptom, pain catastrophizing, and use of medication pre-injury were associated with lower pre-injury EQ-5D-5L summary and EQ-VAS scores. Phase 1 EQ-5D-5L decreases were associated with female sex, no pre-existing body complaints, lack of expectation for a fast recovery, higher ISS, higher injury pain, and neck, spine/back, upper extremity, or lower extremity injuries. Phase 1 EQ-VAS decreases were associated with female sex, lower somatic symptom, fewer comorbidities, lack of expectation for a fast recovery, higher ISS, higher injury pain, neck, spine/back or lower extremity injuries. In phase 2, EQ-5D-5L summary improved most in participants with higher education levels and longer recovery expectations; EQ-VAS improved most in cyclists and patients with longer recovery expectations.

**Conclusions:**

Clinicians should assess and address patients’ recovery expectations early in the care process, as these may significantly influence long-term HRQoL outcomes. Incorporating strategies to support realistic yet positive expectations, such as cognitive-behavioral therapy, structured patient education, and goal-setting programs, may improve recovery experiences. In addition, identifying patients with high pain, or specific injury types may help target early interventions to those at risk of poor HRQoL trajectories.

## Introduction

According to the World Health Organization, 1.3 million people die and up to 50 million individuals are injured each year in road traffic (RT) crashes globally [1]. Advances in medical care and emergency medical services have saved many lives, but many individuals who survive RT injuries face long-term disabilities [2]. RT injuries are typically only considered in terms of their physical impairments, while their psychological impact is often overlooked by both clinicians and researchers. A careful examination of patient-reported health-related quality of life (HRQoL) after RT is one way to acknowledge the full social and psychological burden of road trauma [3,4].

Changes in HRQoL after road trauma can be described as occurring in two phases [5]. In the first phase, which depends on injury severity and typically lasts one to six months,[6] HRQoL scores show a significant decrease compared to population norms or pre-injury level by the time of the first post-injury assessment. In the second phase, there is an increase in HRQoL over the subsequent six months to eight years. Identifying predictors that impede or accelerate recovery in HRQoL following road traffic injury can inform the design of targeted interventions aimed at improving recovery outcomes, thereby helping patients reduce their long-term burden of injury. The study of injured RT populations emphasizes the importance of mental health as an important area of focus. Several studies showed post-injury HRQoL is impaired by post-traumatic stress disorder [7,8], anxiety and depression [8–11]. Other factors associated with reduced HRQoL at follow-up include low recovery expectations [12], and high injury pain [9]. Pain catastrophizing was a significant prognostic factor for poor HRQoL at follow-up [9,11,13]. Some studies found patients with lower extremity and spine injury reported lower HRQoL [14,15]. The correlation between injury severity and HRQoL has been investigated across various populations [7,16–19]. Pre-injury comorbidities have been shown to predict poor HRQoL following RT injury. Therefore, assessing the pre-injury health status of survivors and identifying comorbidities early are recommended to prevent further negative effects of injury [20].

Research on HRQoL after RT has focused on specific groups, which limits its generalizability to all RT survivors. For instance, some studies exclusively investigated motorcyclists [18] or survivors with musculoskeletal injury [10,21]. By including Canadian RT survivors, our study also addresses the limited generalizability of previous RT outcome research which has largely been done outside of North America. The SF-36 (36-Item Short Form Health Survey) is the most commonly used HRQoL measure in RT research [5]. While the SF-36 and its shorter version SF-12 primarily emphasize physical and emotional health subscales, the EQ-5D assesses HRQoL more broadly across multiple dimensions. This multidimensional approach makes the EQ-5D particularly well-suited for capturing the comprehensive impact of injury. To the best of our knowledge, this is the first study in Canada to examine HRQoL trajectories following RT using the EQ-5D-5L instrument, and to include patients across the full spectrum of injury severity.

Our first objective was to pinpoint the potential phases of HRQoL changes (using the 5-level version of EuroQol 5-Dimension (EQ-5D-5L) instrument) over a 12-month period among RT survivors who visited a participating emergency department (ED) in British Columbia (BC), Canada. Furthermore, the second objective was to establish predictors of HRQoL changes over the 12-month period post RT injury.

## Methods

### Study population

This prospective cohort study enrolled survivors of RT from three BC EDs (Vancouver General Hospital, Vancouver; Royal Columbian Hospital, New Westminster; and Kelowna General Hospital, Kelowna) between July 2018 and March 2020. Detailed methods have been previously outlined [22]. RT survivors with all injury severity levels who entered the ED within 24 hours following injuries sustained in a crash with a motor vehicle (cyclists, pedestrians, motorcyclists, and motor vehicle drivers and passengers) were included. Children aged younger than 16 years and non-residents of BC were excluded. Data were collected using medical chart reviews and baseline and follow-up interviews. Interviews were conducted in English for English speakers. Non-English speakers were interviewed through a translator (e.g., family) or multilingual research assistant in Cantonese, French, Korean, Mandarin, Punjabi, and Vietnamese (reflecting the common languages spoken in Greater Vancouver). Participants provided informed written or verbal consent and the study was approved by the University of British Columbia Clinical Research Ethics Board (certificate H18-00284). Participants were compensated for their time with a CAD $15 honorarium after the baseline interview and CAD $10 honoraria for each followup interview.

### HRQoL outcome

We used EQ-5D-5L to assess pre-injury HRQoL at baseline and to assess post-injury HRQoL at 2, 4, 6, and 12 months after injury [23]. The EQ-5D-5L inquires about the individual’s baseline HRQoL status on the day pre-injury. The EQ-5D-5L questionnaire comprises two components: a short descriptive system questionnaire (which assesses health status across five dimensions: mobility, self-care, usual activities, pain/discomfort, and anxiety/depression) and a visual analog scale (EQ-VAS) which assesses overall health of an individual. We calculated an EQ-5D-5L summary score and then standardized according to the Canadian value set [24], with higher scores indicating better health. The EQ-VAS represents a scale for overall health status, spanning from 0 to 100 where 0 signifies the lowest possible health condition and 100 represents the highest possible health condition.

### Potential predictors

At baseline interviews occurring shortly after the injury, we asked patients questions about sociodemographic, psychological, medical, and trauma-related factors. Sociodemographic factors included age, sex, employment status, living situation, education level, ethnicity, years lived in Canada, and pre-existing alcohol, cannabis and/or recreational drug use. Pre-injury psychological factors included somatic symptom severity, pain catastrophizing, and psychological distress. The Patient Health Questionnaire-15 (PHQ-15; 4 weeks pre-injury) was used to assess somatic symptom severity with higher scores indicating greater symptom severity [25]. The Pain Catastrophizing Scale, a validated 13-item 5-point Likert scale, was used to measure pain catastrophizing; higher scores show higher degrees of catastrophic thinking styles [26]. The Patient Health Questionnaire-4 (PHQ-4; 2 weeks pre-injury) evaluated psychological distress with higher scores indicating a greater degree of depression and/or anxiety [27,28]. Pre-injury medical factors included the number of comorbidities, complaints in the injured body area(s), and medication use. The number of comorbidities was determined by patients reporting whether or not they had pre-injury comorbidities, including eye disease, arthritis, diabetes, respiratory disease, heart disease, hypertension, stroke, epilepsy, kidney disease, psychiatric disease, and other diseases. Patients were also asked whether they had experienced any complaints in the affected body area(s) before this injury. Trauma-related factors included ED visit time, road user type, Injury Severity Score (ISS) [29,30], injury pain, injury location, and recovery expectations. Pain reported shortly after the injury was quantified using a visual analog scale (VAS) [31].

### Statistical analysis

**Fig 1:**
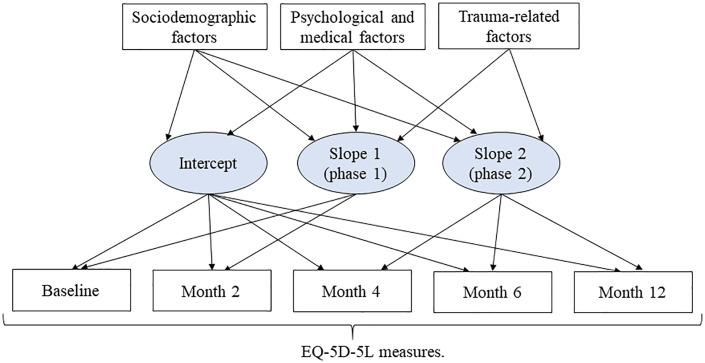
A conceptual framework of piecewise LGCMs for changes in EQ-5D-5L measures following RT.

We explored the presence of multicollinearity among predictors using the Variance Inflation Factor (VIF) to identify highly collinear predictors [32]. At this stage, the variable of ED discharge disposition was deleted from the analysis of this study. Descriptive statistics were utilized to communicate participant characteristics at baseline. To analyze the data, the following two steps were conducted:

First, we analyzed trajectories of EQ-5D-5L summary and EQ-VAS scores using generalized additive mixed models (GAMMs) to describe nonlinear patterns over a 12-month follow-up period. As proposed by White et al., this model assists in visualizing the data and is particularly useful for gaining insights into the development of a subsequent parametric model [33]. However, because GAMMs do not provide explicit parameter estimates for nonlinear relationships between predictors and outcomes, their primary strength lies in exploratory analysis. We compared GAMMs with different random effects structures (random intercept, random slope, and random smooth for time per subject) and selected the model with the lowest Akaike Information Criterion. Also, to further assess model flexibility, we examined the effective degrees of freedom for the smooth terms, which ranged from 3 to 6. Second, a piece-wise latent growth curve model (LGCM) was employed to assess the relationship between baseline covariates encompassing sociodemographic, physiological, medical, and trauma-related factors and both outcomes over time. The piece-wise LGCM assumes linear changes in both outcomes scores over time, with different slopes for the first and second post-injury phases. Fig 1 illustrates the conceptual framework of piece-wise LGCM. As seen, the evolution of longitudinal outcome scores is predicted through three latent growth factors: intercept (mean pre-injury outcome), slope 1 (mean rate of outcome change during phase 1 post-injury), and slope 2 (mean rate of outcome change during phase 2 post-injury). The ratio of $\chi^{2}$ statistic to its degrees of freedom (χ2/χ2df\nulldelimiterspacedf), the root mean square error of approximation (RMSEA), the comparative fit index (CFI), and the Tucker-Lewis index (TLI) were used to assess the overall model fit of LGCM. A χ2/χ2df\nulldelimiterspacedf value of ≤2 [34], RMSEA value <0.08 [35], and CFI and TLI values ≥0.90 indicate good model fitness [35]. A backward elimination algorithm was employed to construct the final piece-wise LGCM, retaining paths significant at the 0.05 level and freeing up nonsignificant paths. We performed 1,000 bootstrap resamples to evaluate the stability of final piece-wise LGCM parameter estimates. Confidence intervals were derived using the bootstrap percentile method (*S3 and S4 Tables in* S1 File). The bootstrap confidence intervals closely matched those from the original model, indicating that the model is stable and not overly sensitive to sample variability. This consistency supports the robustness of our findings.

The comparison of baseline characteristics among participants grouped by follow-up completion status (no follow-up interviews, some follow-up interviews [i.e., one, two, or three], and completion of all follow-up interviews) is presented in *S2 Table in* S1 File. Follow-up completion groups differed significantly in sex, employment status, education level, ethnicity, cannabis use, number of comorbidities, road user type, injury pain, and the presence of neck, spine, or back injuries. These differences suggest that attrition was not completely random, with certain participant subgroups more likely to have incomplete follow-up data. Given that some observed variables are associated with missingness and there is limited uncollected information to explain missing values, the missing at random (MAR) assumption for missing outcome data is reasonable. To address missing data on follow-up outcome variables, we used full information maximum likelihood (FIML) estimation combined with the maximum likelihood estimator within LGCM framework, which assumes a MAR mechanism. We also conducted a sensitivity analysis using the multiple imputation (MI) approach, which can provide valid results under both MAR and missing-not-at-random (MNAR) mechanisms. The MI results were very similar to the FIML results, indicating that our findings are robust and not materially influenced by the MAR assumption. Participants with missing baseline covariate data were excluded from the LGCM. This complete case analysis is considered valid under the reasonable assumption that the missing data are independent of the HRQoL outcome, once baseline covariates are accounted for [36]. As the nature of this analysis is exploratory, *P*-values were not adjusted for multiple comparisons. Data cleaning and analyses were conducted using R version 4.0.5 and Mplus version 7.4. The software code used for data analysis is provided in the supplementary materials file.

## Results

### Description of cohort

**Fig 2:**
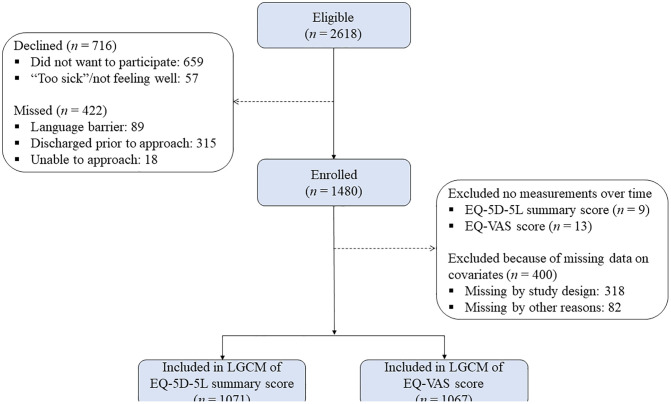
Recruitment flow diagram of the current study.

Of the 1,480 enrolled participants, exclusions for missing repeated measurements and missing covariate data resulted in 1,071 participants for the EQ-5D-5L analysis and 1,067 for the EQ-VAS analysis (Fig 2). Mean age of patients was 43.1 years (ranging from 16 to 103 years), and 54.1% of the sample were males. The majority of RT survivors were drivers (46.1%), 18.9% were pedestrians, 11.8% were cyclists, 15.2% were passengers, and 8.0% were motorcyclists. The majority of participants had minor injuries (median ISS = 3.0, range = 0–66). Table 1 describes other baseline characteristics. The missing data percentage for baseline predictors was low, with less than 3.2% missing, except for five variables of pre-existing alcohol use (21.8%), cannabis use (21.9%), recreational drug use (22.0%), recovery expectations (21.9%), and injury pain (22.3%), because they were not added to the study until March 2019 after recruitment had already started.

**Table 1:** Baseline characteristics of participants (*n *= 1480).

Characteristic	Median [Min-Max]	Number (%) missing responses
**Sociodemographic factors**		
Age in years, mean (SD)	43.1 (18.2)	0 (0.0)
Sex (Male), *n* (%)	800 (54.1)	0 (0.0)
Employment status, *n* (%)		48 (3.2)
Employed	981 (66.3)	
School	154 (10.4)	
Retired	200 (13.5)	
Other	97 (6.6)	
Education level, *n* (%)		17 (1.1)
Less than high school	96 (6.5)	
High school or vocational	569 (38.4)	
University	798 (53.9)	
Ethnicity, *n* (%)		20 (1.4)
White	739 (49.9)	
Asian	364 (24.6)	
Other	357 (24.1)	
Living situation, *n* (%)		23 (1.6)
Alone	351 (23.7)	
With others	1106 (74.7)	
Years lived in Canada, *n* (%)		19 (1.3)
> 10 years	1252 (84.6)	
≤ 10 years	209 (14.1)	
Pre-existing alcohol use (Yes)^a^, *n* (%)	740 (50.0)	323 (21.8)
Pre-existing cannabis use (Yes)^a^, *n* (%)	297 (20.1)	324 (21.9)
Pre-existing recreational drug use (Yes)^a^, *n* (%)	53 (3.6)	326 (22.0)
**Psychological and medical factors**		
Somatic symptom severity (PHQ-15)	2.1 [0.0-25.0]	47 (3.2)
Pain catastrophizing	4.0 [0.0-52.0]	45 (3.0)
Psychological distress (PHQ-4)	0.0 [0.0-12.0]	5 (0.3)
Pre-injury comorbidities number	1.0 [0.0-9.0]	0 (0.0)
Pre-injury body complaints (Yes), *n* (%)	319 (21.6)	14 (0.9)
Pre-injury medication use (Yes), *n* (%)	615 (41.6)	0 (0.0)
**Trauma-related factors**		
Time of ED visit, *n* (%)		0 (0.0)
Daytime	966 (65.3)	
Nighttime	514 (34.7)	
Road user type, *n* (%)		0 (0.0)
Driver	683 (46.1)	
Motor vehicle passenger	225 (15.2)	
Motorcyclist	118 (8.0)	
Pedestrian	280 (18.9)	
Cyclist	174 (11.8)	
ISS	3.0 [0.0-66.0]	1 (0.1)
Injury pain (VAS)^a^, mean (SD)	5.3 (2.4)	330 (22.3)
Injury location, *n* (%)		
Head (Yes)	567 (38.3)	0 (0.0)
Neck (Yes)	558 (37.7)	0 (0.0)
Torso (Yes)	567 (38.3)	0 (0.0)
Spine/back (Yes)	501 (33.9)	0 (0.0)
Upper extremity (Yes)	744 (50.3)	0 (0.0)
Lower extremity (Yes)	705 (47.6)	0 (0.0)
Recovery expectations^a^, *n* (%)		324 (21.9)
Less than 1 month	395 (26.7)	
More than 1 month	263 (17.8)	
No idea	498 (33.6)	

SD: standard deviation; PHQ-15: Patient Health Questionnaire-15; PHQ-4: Patient Health Questionnaire-4; ED: emergency department; ISS: injury severity score; VAS: visual analog scale.

^a^Variable was added to the study protocol in March 2019.

### GAMM of EQ-5D-5L measures

**Fig 3:**
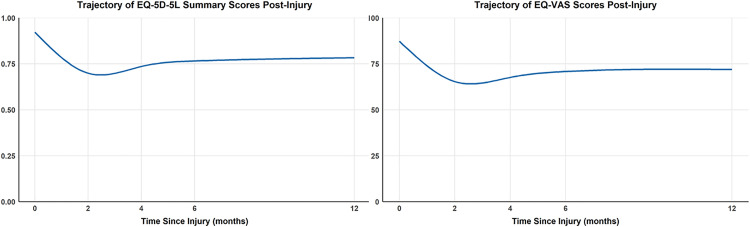
Estimated trajectories of EQ-5D-5L summary score and EQ-VAS using generalized additive mixed model.

Of the 1,071 participants with baseline EQ-5D-5L summary scores, available responses decreased to 770 (71.9%) at 2 months, 681 (63.6%) at 4 months, 669 (62.5%) at 6 months, and 634 (59.2%) at 12 months, reflecting missing data rates of approximately 28%, 36%, 38%, and 41% respectively (Table 2). Similarly, among the 1,067 participants with baseline EQ-VAS scores, completed responses were 768 (72.0%) at 2 months, 674 (63.2%) at 4 months, 657 (61.6%) at 6 months, and 630 (59.1%) at 12 months. Fig 3 provides the findings of the GAMM for EQ-5D-5L summary and EQ-VAS scores. As seen, the estimated trajectory of EQ-5D-5L summary and EQ-VAS scores significantly decreased compared to the baseline at 2-months (phase 1; worsening), and then started to increase after 2-months (phase 2; improving), but did not return to baseline values at 12-months.

**Table 2:** Estimated summary statistics of EQ-5D-5L measures over time using a generalized additive mixed model.

Outcomes	Number	Mean (SE)
**EQ-5D-5L summary score (*n *= 1071)**		
Baseline	1069	0.92 (0.0005)
Month 2	770	0.69 (0.0006)
Month 4	681	0.73 (0.0006)
Month 6	669	0.76 (0.0006)
Month 12	634	0.78 (0.0007)
**EQ-VAS score (*n *= 1067)**		
Baseline	1065	87.3 (0.038)
Month 2	768	65.4 (0.045)
Month 4	674	67.7 (0.048)
Month 6	657	70.9 (0.049)
Month 12	630	71.9 (0.048)

SD: standard error; EQ-5D-5L: European Quality of Life-5 Dimensions; EQ-VAS: EQ-5D-5L visual analog scale.

Table 2 presents summary scores for EQ-5D-5L and EQ-VAS outcomes over time based on GAMM estimates. The mean pre-injury scores for EQ-5D-5L and EQ-VAS were 0.92 and 87.34 points, respectively. During the first phase, the mean reduction in EQ-5D-5L was −0.22, which exceeds the minimally important difference (MID) of 0.01–0.41 reported for injured populations [37], indicating a clinically meaningful decline in HRQoL. Similarly, the mean reduction in EQ-VAS was −21.9 points, which is greater than the MID of 0.04–51 points [37], reflecting a substantial perceived deterioration in overall health status. In the second phase, EQ-5D-5L and EQ-VAS scores improved by 0.084 and 6.5 points, respectively, changes that fall within the MID range and suggest meaningful recovery.

### Piece-wise LGCM of EQ-5D-5L measures

Based on the fit criteria, the piece-wise LGCM for the EQ-5D-5L summary and EQ-VAS scores fits the data very well (*S1 Table in* S1 File). Tables 3,4 provide the findings of the piece-wise LGCM for EQ-5D-5L summary and EQ-VAS scores, respectively. Participants who lived alone, lived for more than 10 years in Canada, those with White ethnicity, higher somatic symptom severity, pain catastrophizing, psychological distress, pre-injury complaints in the injured body, and medication use pre-injury reported lower mean pre-injury EQ-5D-5L summary scores. In the first phase, females and participants with no pre-injury body complaints, lack of expectation for a fast recovery, higher ISS, higher injury pain, as well as those with neck, spine/back, upper extremity, or lower extremity injuries reported a sharper decrease in EQ-5D-5L summary scores. Compared to employed participants, students had less decrease in EQ-5D-5L summary scores in the first phase. During the second phase, individuals with higher levels of education and longer recovery expectations showed greater improvements in EQ-5D-5L summary scores. During the second phase, patients with higher ISS and who sustained upper extremity or lower extremity injuries reported larger improvements in EQ-5D-5L summary scores. Males, and participants with White ethnicity, higher somatic symptom severity, pain catastrophizing, more comorbidities, and pre-injury medication use reported lower pre-injury EQ-VAS scores. In phase one, females and participants with lower pre-injury somatic symptom severity, less comorbidities, lack of expectation for a fast recovery, higher ISS, higher injury pain, as well as those with neck, spine/back or lower extremity injuries reported a sharper decrease in EQ-VAS scores. In the second phase, participants with longer recovery expectations reported greater improvements in EQ-VAS scores. Cyclists reported more improvement in EQ-VAS scores compared to motor vehicle drivers.

**Table 3:** Results of final piece-wise LGCM for assessing the concurrent effect of predictors on changes in EQ-5D-5L summary score (*n *= 1071).

	Intercept (pre-injury)		Slope 1 (phase 1 post-injury: worsening)		Slope 2 (phase 2 post-injury: improving)	
	Estimate (SE)	P-value	Estimate (SE)	P-value	Estimate (SE)	P-value
Intercept	0.964 (0.004)	<0.001	0.036 (0.009)	<0.001	0.001 (0.001)	0.773
**Sociodemographic factors**						
Sex (Ref: Male)						
Female	**—**	**—**	−0.016 (0.006)	0.007	**—**	**—**
Employment status (Ref: Employed)						
School	**—**	**—**	0.019 (0.008)	0.014	**—**	**—**
Retired	**—**	**—**	0.003 (0.008)	0.707	**—**	**—**
Other	**—**	**—**	0.003 (0.013)	0.811	**—**	**—**
Education level (Ref: University)						
Less than high school	**—**	**—**	**—**	**—**	−0.002 (0.001)	0.024
High school or vocational	**—**	**—**	**—**	**—**	−0.008 (0.004)	0.058
Ethnicity (Ref: White)						
Asian	−0.001 (0.005)	0.775	**—**	**—**	**—**	**—**
Other	0.009 (0.004)	0.016	**—**	**—**	**—**	**—**
Living situation (Ref: With others)						
Alone	−0.011 (0.005)	0.015	**—**	**—**	**—**	**—**
Years lived in Canada (Ref: > 10 years)						
≤ 10 years	0.008 (0.003)	0.009	**—**	**—**	**—**	**—**
**Psychological and medical factors**						
Somatic symptom severity (PHQ-15)	−0.004 (0.001)	<0.001			**—**	**—**
Pain catastrophizing	−0.001 (0.0001)	0.003	**—**	**—**	**—**	**—**
Psychological distress (PHQ-4)	−0.01 (0.002)	<0.001	**—**	**—**	**—**	**—**
Pre-injury body complaints (Ref: No)						
Yes	−0.033 (0.006)	<0.001	0.020 (0.008)	0.010	**—**	**—**
Pre-injury medication use (Ref: No)						
Yes	−0.011 (0.003)	0.002	**—**	**—**	**—**	**—**
**Trauma-related factors**						
ISS	**†**	**†**	−0.003 (0.001)	<0.001	0.0001 (0.0001)	<0.001
	**†**	**†**	−0.010 (0.001)	<0.001	**—**	**—**
Neck injury (Ref: No)						
Yes	**†**	**†**	−0.017 (0.006)	0.007	**—**	**—**
Spine/back injury (Ref: No)						
Yes	**†**	**†**	−0.019 (0.007)	0.004	**—**	**—**
Upper extremity injury (Ref: No)						
Yes	**†**	**†**	−0.013 (0.007)	0.047	0.004 (0.001)	0.004
Lower extremity injury (Ref: No)						
Yes	**†**	**†**	−0.026 (0.007)	<0.001	0.003 (0.001)	0.011
Recovery expectations (Ref: Less than 1 month)						
More than 1 month	**†**	**†**	−0.043 (0.010)	<0.001	0.004 (0.002)	0.024
No idea	**†**	**†**	−0.037 (0.007)	<0.001	0.001 (0.001)	0.393

**—**Not significant in the final model.

**†** Not entered in the first model.

LGCM: latent growth curve model; EQ-5D-5L: European Quality of Life-5 Dimensions; RT: road trauma; Ref: reference; SE: standard error; PHQ-15: Patient Health Questionnaire-15; PHQ-4: Patient Health Questionnaire-4; ISS: injury severity score; VAS: visual analog scale.

**Table 4:** Results of final piece-wise LGCM for assessing the concurrent effect of predictors on changes in EQ-VAS score (*n *= 1067).

	Intercept (pre-injury)		Slope 1 (phase 1 post-injury: worsening)		Slope 2 (phase 2 post-injury: improving)	
	Estimate (SE)	P-value	Estimate (SE)	P-value	Estimate (SE)	P-value
Intercept	93.22 (0.66)	<0.001	−2.92 (0.84)	0.001	0.99 (0.20)	<0.001
**Sociodemographic factors**						
Sex (Ref: Male)						
Female	1.91 (0.69)	<0.001	−1.69 (0.61)	0.006	**—**	**—**
Ethnicity (Ref: White)						
Asian	−0.13 (0.75)	0.862	**—**	**—**	**—**	**—**
Other	2.17 (0.76)	<0.001	**—**	**—**	**—**	**—**
**Psychological and medical factors**						
Somatic symptom severity (PHQ-15)	−1.12 (0.13)	<0.001	0.30 (0.11)	0.007	**—**	**—**
Pain catastrophizing	−0.18 (0.04)	<0.001	**—**	**—**	**—**	**—**
Pre-injury comorbidities number	−1.42 (0.33)	<0.001	0.54 (0.24)	0.026	**—**	**—**
Pre-injury medication use (Ref: No)						
Yes	−2.42 (0.66)	<0.001	**—**	**—**	**—**	**—**
**Trauma-related factors**						
Road user type (Ref: Driver)						
Cyclist	**†**	**†**	**—**	**—**	0.36 (0.16)	0.028
Motorcyclist	**†**	**†**	**—**	**—**	0.23 (0.20)	0.253
Motor vehicle passenger	**†**	**†**	**—**	**—**	0.23 (0.17)	0.189
Pedestrian	**†**	**†**	**—**	**—**	−0.29 (0.20)	0.137
ISS	**†**	**†**	−0.13 (0.04)	0.002	**—**	**—**
Injury pain (VAS)	**†**	**†**	−0.66 (0.11)	<0.001	**—**	**—**
Neck injury (Ref: No)		**†**				
Yes	**†**	**†**	−1.69 (0.59)	0.004	**—**	**—**
Spine/back injury (Ref: No)		**†**				
Yes	**†**	**†**	−1.45 (0.60)	0.015	**—**	**—**
Lower extremity injury (Ref: No)		**†**				
Yes	**†**	**†**	−1.17 (0.56)	0.037	**—**	**—**
Recovery expectations (Ref: Less than 1 month)		**†**				
More than 1 month	**†**	**†**	−4.70 (0.85)	<0.001	0.56 (0.18)	0.003
No idea	**†**	**†**	−4.17 (0.70)	<0.001	0.20 (0.15)	0.186

**—**Not significant in the final model.

**†** Not entered in the first model.

LGCM: latent growth curve model; EQ-VAS: EQ-5D-5L visual analog scale; RT: road trauma; Ref: reference; SE: standard error; PHQ-15: Patient Health Questionnaire-15; PHQ-4: Patient Health Questionnaire-4; VAS: visual analog scale.

## Discussion

This comprehensive prospective cohort study of Canadian RT survivors provides robust insights into the nonlinear trajectories of HRQoL over 12 months post-injury, leveraging the EQ-5D-5L instrument and a diverse range of validated predictors to inform targeted rehabilitation strategies. This study revealed that the trajectory of EQ-5D-5L summary scores and EQ-VAS scores over a year following RT injury was nonlinear. There were two phases, one indicating a decline in EQ-5D-5L measures from baseline to 2-months, and the other showing improvement from 2-months to 12-months, respectively. This result adds to the already robust evidence demonstrating that impaired HRQoL following RT injury is common, even in cases of minor injuries, highlighting the necessity of rehabilitation interventions across all levels of injury. In agreement with our results, Gopinath et al. demonstrated that both EQ-5D-5L summary and EQ-VAS scores decreased after non-catastrophic RT injury compared to pre-injury levels and did not return to pre-injury scores by 12 months [9]. Another Australian study indicated that EQ-5D-5L summary scores significantly declined after non-catastrophic RT injuries compared to population norms among cyclists and car occupants (drivers and passengers) [13]. Cyclists returned to population norms at 12 months after injury, whereas car occupants did not. Gopinath et al. demonstrated that among both older (65 years and above) and younger (18–64 years) participants, EQ-VAS scores did not return to Australian population norms within the corresponding age groups even 24 months after a mild to moderate musculoskeletal injury [38].

Our findings are consistent with our previous work using the SF-12 instrument, where we also identified a similar nonlinear HRQoL trajectory following RT injury, characterized by an early decline followed by partial recovery over time [39]. This research highlights the wide spectrum of pre-injury factors that are associated both cross-sectionally and prospectively with the HRQoL of RT survivors. These factors may help clinicians identify patients at risk of long-term impaired HRQoL at the time of admission. Once risk factors are identified, clinicians may be able to design interventions that address those factors or that target individuals at risk of poor outcome. Living alone or for more than 10 years in Canada, White ethnicity, higher somatic symptom severity, pain catastrophizing, psychological distress, pre-injury complaints in the injured body part, and pre-injury medication use were associated with lower mean pre-injury EQ-5D-5L summary scores. Being male, White, having higher somatic symptom severity, pain catastrophizing, more comorbidities, and pre-injury medication use were associated with lower pre-injury EQ-VAS scores. The association between living alone and poorer pre-injury HRQoL aligns with previous research demonstrating that social isolation and reduced social support are linked to poorer HRQoL [40]. The finding that participants of White ethnicity reported lower pre-injury HRQoL contrasts with some studies where minority ethnic groups reported poorer health status [41], but is in line with other research [42]. Our model identified certain prospective associations that differ from previous findings. First, not having pre-injury complaints in the injured body area was associated with more reduction in EQ-5D-5L summary scores post-injury. Second, lower pre-injury somatic symptom severity and fewer comorbidities were associated with more reduction in EQ-VAS scores post-injury. These findings are likely due to the fact that patients in our sample who had no body complaints, lower pre-injury somatic symptom severity, and fewer comorbidities had higher baseline HRQoL, allowing for more reductions in HRQoL following injury. These results could also be explained by the possibility that RT survivors with less pre-injury experience of body complaints, somatic symptoms, or comorbidities may perceive their post-injury symptoms as more severe compared to RT survivors with more pre-injury exposure to these factors. In other words, RT survivors who experience these factors before the injury may be better able to adapt to the injury challenges.

Consistent with previous studies [9,10,18], this study found that female participants were more vulnerable to impaired HRQoL following RT. This may be explained by variations in the perception or expression of pain, discomfort, and bodily symptoms between males and females [43]. However, similar to earlier research reporting that, on average, women had higher HRQoL than men in non-injured populations, our sample also showed that females had higher HRQoL prior to injury [44]. A similar report regarding ISS and injury pain was also found by Khati et al. [15] and Fitzharris et al. [45] following RT injury. Similar findings of increased vulnerability associated with neck, spine/back, lower extremity and upper extremity injuries have also been reported by other cohort studies following RT injury [9,14,15]. Consistent with findings from Australian research [19], our study revealed that patients who had fast recovery expectations experienced less reduction in HRQoL during the initial phase following injury. In our study patients with lack of fast recovery expectation, higher ISS, lower extremity and upper extremity injuries had higher recovery during the second phase because they had more room for improvement. Consistent with previous studies [16,46], our results highlight that shorter education is associated with poorer recovery in HRQoL. It also makes sense that being a cyclist is associated with a faster rate of HRQoL recovery, potentially reflecting healthier lifestyles and better pre-injury functioning [47]; however, further research is needed to confirm this.

The strengths of this study include its design, which involved five waves of data collection, a wide range of pre-injury potential predictors using validated scales, and the consideration of all types of road users and injury severity levels. However, there are some limitations to this study. First, a self-reported questionnaire was used to measure pre-injury characteristics (HRQoL, somatic symptom severity, pain catastrophizing, psychological distress), which can lead to recall bias. However, we attempted to minimize this by conducting baseline interviews within seven days of the injury in most cases. Second, older patients and those with severe pain may be less likely to participate in this study, which can lead to selection bias. Additionally, our design excluded RT survivors who did not seek hospital treatment. Those who were quickly discharged from the ED may have also been overlooked in the study. Third, while most measures were obtained using validated scales, those assessing pre-existing substance use, medication use, and comorbidities were not validated, and related results should be interpreted with caution. Fourth, the absence of important socioeconomic variables such as household income, urban versus rural residence, and health insurance coverage limits our ability to fully capture HRQoL predictors. Future studies should incorporate these variables to provide a more comprehensive understanding of HRQoL determinants in this population. Fifth, *P*-values in the LGCM were not adjusted for multiple comparisons, so the results need to be interpreted with caution. Finally, causality cannot be established in our study as it was observational. Future studies should test interventions targeting factors such as recovery expectations to enhance HRQoL outcomes among RT survivors.

## Conclusions

In summary, this cohort study of patients with predominantly minor RT injuries showed an initial decline in HRQoL at 2 months post-injury, followed by improvement by 12 months. Several factors, such as sex, education, psychological distress, pain catastrophizing, comorbidities, recovery expectations, ISS, injury pain, and injury location, were associated with HRQoL both cross-sectionally and over time. Notably, recovery expectations emerged as a potentially modifiable factor influencing HRQoL trajectories. Clinicians treating RT patients should assess and address recovery expectations, as modifying poor expectations may improve patient outcomes. Interventions such as cognitive-behavioral therapy, structured patient education, and goal-setting programs warrant further research to determine their effectiveness in enhancing rehabilitation strategies and preventing substantial HRQoL reductions following RT.

## Supporting information

S1 File Supporting Information. This file contains six supplementary tables providing additional results: **S1 Table.** Model fit indices for the final piecewise LGCMs. **S2 Table.** Comparison of baseline characteristics between participants completing no follow-up interviews, some follow-up interviews (i.e., one, two, or three), and all follow-up interviews (n = 1480). **S3 Table.** Bootstrap results of final piecewise LGCM for assessing the concurrent effect of predictors on changes in EQ-5D-5L summary score (n = 1071). **S4 Table.** Bootstrap results of final piecewise LGCM for assessing the concurrent effect of predictors on changes in EQ-VAS score (n = 1067). **S5 Table.** Results of final piecewise LGCM for assessing the concurrent effect of predictors on changes in EQ-5D-5L summary score in imputed data (n = 1071). **S6 Table.** Results of final piecewise LGCM for assessing the concurrent effect of predictors on changes in EQ-VAS score in imputed data (n = 1067).
(DOCX)
