# Supplementary material for: Longitudinal modeling of health-related quality of life trajectories over 12 months following road trauma
Source: PLoS One. 2025 Nov 21;20(11):e0336144. doi: 10.1371/journal.pone.0336144 (PMC12637921; doi:10.1371/journal.pone.0336144)
Supplement: S1 File — This file contains six supplementary tables providing additional results: S1 Table. Model fit indices for the final piecewise LGCMs. S2 Table. Comparison of baseline characteristics between participants completing no follow-up interviews, some follow-up interviews (i.e., one, two, or three), and all follow-up interviews (n = 1480). S3 Table. Bootstrap results of final piecewise LGCM for assessing the concurrent effect of predictors on changes in EQ-5D-5L summary score (n = 1071). S4 Table. Bootstrap results of final piecewise LGCM for assessing the concurrent effect of predictors on changes in EQ-VAS score (n = 1067). S5 Table. Results of final piecewise LGCM for assessing the concurrent effect of predictors on changes in EQ-5D-5L summary score in imputed data (n = 1071). S6 Table. Results of final piecewise LGCM for assessing the concurrent effect of predictors on changes in EQ-VAS score in imputed data (n = 1067). (DOCX) [file pone.0336144.s001.docx]

**Supplementary Material**

**Longitudinal modeling of health-related quality of life trajectories over 12 months following road trauma**

**1. Latent growth curve model (****LGCM)**

Supplementary *S1* *Tables* show the fit criteria of piecewise LGCMs.

| **S1 Table. Model fit indices for the final piecewise LGCMs.** | | | | |
| --- | --- | --- | --- | --- |
|  | $\chi^{2}/df$ | RMSEA (90% CI) | CFI | TLI |
| **EQ-5D-5L summary score (*n* = 1071)** | 97.05/92 | 0.007 (0.000, 0.018) | 0.99 | 0.99 |
| **EQ-VAS scores (*n* = 1067)** | 52.17/71 | 0.000 (0.000, 0.000) | 1.00 | 1.00 |
| LGCM: latent growth curve model; df: degree of freedom; RMSEA: root mean square error of approximation; CI: confidence interval; CFI: comparative fit index; TLI: Tucker-Lewis index. | | | | |

**2. Missingness**

The comparison of baseline characteristics across follow-up completion groups (no follow-up interviews, some follow-up interviews (i.e., one, two or three), and all follow-up interviews) is presented in supplementary *S2 Table* below. Follow-up completion groups differed according to sex, employment status, education level, ethnicity, cannabis use, number of comorbidities, road user type, injury pain, and neck and spine or back injuries. Given that some variables are associated with missingness and there is little uncollected information that would explain missing values, the missing at random assumption is reasonable in missing data on outcomes.

| **S2 Table. Comparison of baseline characteristics between participants completing no follow-up interviews, some follow-up interviews (i.e., one, two or three), and all follow-up interviews (*n* = 1480).** | | | | | | |
| --- | --- | --- | --- | --- | --- | --- |
|  | **Characteristic** | | No follow-up  (*n* = 263) | Some follow-up  (*n* = 512) | All follow-up  (*n* = 705) | P-value^a^ |
| **Sociodemographic factors** | | |  |  |  |  |
|  | Age in years | | 42.2 (18.0) | 42.9 (17.9) | 43.6 (18.6) | 0.573 |
|  | Sex | |  |  |  |  |
|  |  | Male | 164 (62.4) | 287 (56.1) | 349 (49.5) | 0.001 |
|  |  | Female | 99 (37.6) | 225 (43.9) | 356 (50.5) |  |
|  | Employment status | |  |  |  |  |
|  |  | Employed | 166 (68.6) | 342 (69.4) | 468 (67.8) | 0.033 |
|  |  | School | 23 (9.5) | 51 (10.2) | 80 (11.6) |  |
|  |  | Retired | 28 (11.6) | 63 (12.6) | 109 (15.8) |  |
|  |  | Other | 25 (10.3) | 39 (7.8) | 33 (4.8) |  |
|  | Living situation | |  |  |  |  |
|  |  | Alone | 70 (29.0) | 118 (23.0) | 163 (23.2) | 0.144 |
|  |  | With others | 171 (71.0) | 394 (77.0) | 541 (76.8) |  |
|  | Education level | |  |  |  |  |
|  |  | Less than high school | 24 (9.7) | 46 (9.0) | 26 (3.7) | <0.001 |
|  |  | High school or vocational | 115 (46.6) | 229 (44.7) | 225 (32.0) |  |
|  |  | University | 108 (43.7) | 237 (46.3) | 453 (64.3) |  |
|  | Ethnicity | |  |  |  |  |
|  |  | White | 115 (47.2) | 232 (45.4) | 392 (55.6) | 0.001 |
|  |  | Asian | 76 (31.1) | 129 (25.2) | 159 (22.6) |  |
|  |  | Other | 53 (21.7) | 150 (29.4) | 154 (21.8) |  |
|  | Years lived in Canada | |  |  |  |  |
|  |  | > 10 years | 207 (84.5) | 436 (85.2) | 609 (86.5) | 0.670 |
|  |  | $\leq$ 10 years | 38 (15.5) | 76 (14.8) | 95 (13.5) |  |
|  | Pre-existing alcohol use | |  |  |  |  |
|  |  | Yes | 119 (60.7) | 250 (61.0) | 371 (67.3) | 0.074 |
|  |  | No | 77 (39.3) | 160 (39.0) | 180 (32.7) |  |
|  | Pre-existing cannabis use | |  |  |  |  |
|  |  | Yes | 59 (30.1) | 119 (29.0) | 119 (21.6) | 0.010 |
|  |  | No | 137 (69.9) | 291 (71.0) | 431 (78.4) |  |
|  | Pre-existing recreational drug use | |  |  |  |  |
|  |  | Yes | 15 (7.7) | 19 (4.6) | 19 (3.5) | 0.052 |
|  |  | No | 180 (92.3) | 390 (95.4) | 531 (96.5) |  |

**Table S2 Continued.**

|  |  | |  |  |  |  |
| --- | --- | --- | --- | --- | --- | --- |
| **Characteristic** | | | No follow-up  (*n* = 263) | Some follow-up  (*n* = 512) | All follow-up  (*n* = 705) | P-value^a^ |
| **Psychological and medical factors** | | | |  |  |  |
|  | Somatic symptom severity (PHQ-15) | | 3.2 (3.9) | 3.4 (3.8) | 3.2 (3.4) | 0.600 |
|  | Pain catastrophizing | | 8.2 (10.7) | 7.2 (9.5) | 7.2 (8.7) | 0.382 |
|  | Psychological distress (PHQ-4) | | 1.2 (2.6) | 1.2 (2.2) | 1.1 (2.0) | 0.097 |
|  | Pre-injury comorbidities number | | 0.7 (1.0) | 0.9 (1.2) | 1.1 (1.3) | 0.017 |
|  | Pre-injury body complaints | |  |  |  |  |
|  |  | Yes | 46 (17.8) | 105 (20.7) | 168 (24.0) | 0.086 |
|  |  | No | 213 (82.2) | 403 (79.3) | 531 (76.0) |  |
|  | Pre-injury medication use | |  |  |  |  |
|  |  | Yes | 97 (36.9) | 209 (40.8) | 309 (43.8) | 0.137 |
|  |  | No | 166 (63.1) | 303 (59.2) | 396 (56.2) |  |
| **Trauma-related factors** | | |  |  |  |  |
|  | Time of ED visit | |  |  |  |  |
|  |  | Daytime | 169 (64.3) | 319 (62.3) | 478 (67.8) | 0.129 |
|  |  | Nighttime | 94 (35.7) | 193 (37.7) | 227 (32.2) |  |
|  | Road user type | |  |  |  |  |
|  |  | Driver | 116 (44.1) | 249 (48.6) | 318 (45.1) | 0.013 |
|  |  | Motor vehicle passenger | 39 (14.8) | 90 (17.6) | 96 (13.6) |  |
|  |  | Motorcyclist | 21 (8.0) | 37 (7.2) | 60 (8.5) |  |
|  |  | Pedestrian | 66 (25.1) | 82 (16.0) | 132 (18.7) |  |
|  |  | Cyclist | 21 (8.0) | 54 (10.5) | 99 (14.0) |  |
|  | ISS | | 6.9 (8.8) | 7.3 (10.2) | 6.9 (8.9) | 0.534 |
|  | Injury pain (VAS) | | 5.6 (2.4) | 5.6 (2.3) | 5.1 (2.4) | <0.001 |
|  | Injury location | |  |  |  |  |
|  |  | Head (Yes) | 106 (40.3) | 200 (39.1) | 261 (37.0) | 0.588 |
|  |  | Neck (Yes) | 100 (38.6) | 215 (42.0) | 243 (34.5) | 0.028 |
|  |  | Torso (Yes) | 95 (36.1) | 180 (35.2) | 292 (41.4) | 0.062 |
|  |  | Spine/back (Yes) | 89 (33.8) | 194 (37.8) | 218 (30.9) | 0.040 |
|  |  | Upper extremity (Yes) | 125 (47.5) | 250 (48.8) | 369 (52.3) | 0.297 |
|  |  | Lower extremity (Yes) | 133 (50.6) | 237 (46.3) | 335 (47.5) | 0.526 |
| Recovery expectations | | |  |  |  |  |
|  |  | Less than 1 month | 57 (29.1) | 147 (35.8) | 191 (34.8) | 0.213 |
|  |  | More than 1 month | 48 (24.5) | 81 (19.7) | 134 (24.4) |  |
|  |  | No idea | 91 (46.4) | 183 (44.5) | 224 (40.8) |  |
| Data are mean (standard deviation) for continuous variables and number (percent) for categorical variables.  PHQ-15: Patient Health Questionnaire-15; PHQ-4: Patient Health Questionnaire-4; ED: emergency department; ISS: Injury severity score; VAS: visual analog scale.  ^a^ P-values obtained through chi-squared test for categorical variables and ANOVA or Kruskal‐Wallis tests for continuous variables comparing across follow-up completion groups. | | | | | | |

**3. External validation**

| **S3 Table. Bootstrap results of final piece-wise LGCM for assessing the concurrent effect of predictors on changes in EQ-5D-5L summary score (*n* = 1071).** | | | | | | | | |
| --- | --- | --- | --- | --- | --- | --- | --- | --- |
|  | | | Intercept  (pre-injury) | | Slope 1  (phase 1 post-injury: worsening) | | Slope 2  (phase 2 post-injury: improving) | |
|  | | | **95% Bootstrap CI** |  | **95% Bootstrap CI** |  | **95% Bootstrap CI** |  |
| **Sociodemographic factors** | | |  |  |  |  |  |  |
|  | Sex (Ref: Male) | |  |  |  |  |  |  |
|  |  | Female | **—** |  | (-0.027, - 0.004) |  | **—** |  |
|  | Employment status (Ref: Employed) | |  |  |  |  |  |  |
|  |  | School | **—** |  | (0.003, 0.033) |  | **—** |  |
|  |  | Retired | **—** |  | (-0.022, 0.028) |  | **—** |  |
|  |  | Other | **—** |  | (-0.022, 0.028) |  | **—** |  |
|  | Education level (Ref: University) | |  |  |  |  |  |  |
|  |  | Less than high school | **—** |  | **—** |  | (-0.005, 0.000) |  |
|  |  | High school or vocational | **—** |  | **—** |  | (-0.016, 00.000) |  |
|  | Ethnicity (Ref: White) | |  |  |  |  |  |  |
|  |  | Asian | (-0.012, 0.008) |  | **—** |  | **—** |  |
|  |  | Other | (0.001, 0.016) |  | **—** |  | **—** |  |
|  | Living situation (Ref: With others) | |  |  |  |  |  |  |
|  |  | Alone | (-0.020, - 0.002) |  | **—** |  | **—** |  |
|  | Years lived in Canada (Ref: >10 years) | |  |  |  |  |  |  |
|  |  | ≤10 years | (0.002, 0.015) |  | **—** |  | **—** |  |
| **Psychological and medical factors** | | |  |  |  |  |  |  |
|  | Somatic symptom severity (PHQ-15) | | (-0.006, - 0.003) |  |  |  | **—** |  |
|  | Pain catastrophizing | | (-0.001, 0.000) |  | **—** |  | **—** |  |
|  | Psychological distress (PHQ-4) | | (-0.013, - 0.006) |  | **—** |  | **—** |  |
|  | Pre-injury body complaints (Ref: No) | |  |  |  |  |  |  |
|  | Yes | | (-0.048, - 0.022) |  | (0.005, 0.036) |  | **—** |  |
|  | Pre-injury medication use (Ref: No) | |  |  |  |  |  |  |
|  | Yes | | (-0.018, - 0.004) |  | **—** |  | **—** |  |
| **Trauma-related factors** | | |  |  |  |  |  |  |
|  | ISS | | **†** |  | -0.003 (0.001) |  | (0.000, 0.0002) |  |
|  | Injury pain (VAS) | | **†** |  | -0.010 (0.001) |  | **—** |  |
|  | Neck injury (Ref: No) | |  |  |  |  |  |  |
|  |  | Yes | **†** |  | (-0.030, - 0.004) |  | **—** |  |
|  | Spine/back injury (Ref: No) | |  |  |  |  |  |  |
|  |  | Yes | **†** |  | (-0.033, - 0.006) |  | **—** |  |
|  | Upper extremity injury (Ref: No) | |  |  |  |  |  |  |
|  |  | Yes | **†** |  | (-0.026, 0.001) |  | (0.001, 0.006) |  |
|  | Lower extremity injury (Ref: No) | |  |  |  |  |  |  |
|  |  | Yes | **†** |  | (-0.040, - 0.014) |  | (0.001, 0.005) |  |
|  | Recovery expectations (Ref: Less than 1 month) | |  |  |  |  |  |  |
|  |  | More than 1 month | **†** |  | (-0.063, - 0.025) |  | (0.000, 0.007) |  |
|  |  | No idea | **†** |  | (-0.052, - 0.022) |  | (-0.001, 0.004) |  |
|  | **—**Not significant in the final model.  **†** Not entered in the first model.  LGCM: latent growth curve model; EQ-5D-5L: European Quality of Life-5 Dimensions; RT: road trauma; Ref: reference; SE: standard error; PHQ-15: Patient Health Questionnaire-15; PHQ-4: Patient Health Questionnaire-4; ISS: injury severity score; VAS: visual analog scale. | | | | | | | |

| **S4 Table. Bootstrap results of final piece-wise LGCM for assessing the concurrent effect of predictors on changes in EQ-VAS score (*n* = 1067).** | | | | | | | | |  |
| --- | --- | --- | --- | --- | --- | --- | --- | --- | --- |
|  | | | Intercept  (pre-injury) | | Slope 1  (phase 1 post-injury: worsening) | | Slope 2  (phase 2 post-injury: improving) | |  |
|  | | | **95% Bootstrap CI** |  | **95% Bootstrap CI** |  | **95% Bootstrap CI** |  |  |
| **Sociodemographic factors** | | |  |  |  |  |  |  |  |
|  | Sex (Ref: Male) | |  |  |  |  |  |  |  |
|  |  | Female | (0.647, 3.166) |  | (-2.912, -0.499) |  | **—** |  |  |
|  | Ethnicity (Ref: White) | |  |  |  |  |  |  |  |
|  |  | Asian | (-1.627, 1.219) |  | **—** |  | **—** |  |  |
|  |  | Other | (0.761, 3.803) |  | **—** |  | **—** |  |  |
| **Psychological and medical factors** | | |  |  |  |  |  |  |  |
|  | Somatic symptom severity (PHQ-15) | | (-1.368, -0.868) |  | (0.101, 0.513) |  | **—** |  |  |
|  | Pain catastrophizing | | (-0.279, -0.108) |  | **—** |  | **—** |  |  |
|  | Pre-injury comorbidities number | | (-2.105, -0.740) |  | (0.043, 1.055) |  | **—** |  |  |
|  | Pre-injury medication use (Ref: No) | |  |  |  |  |  |  |  |
|  |  | Yes | (-3.850, -1.156) |  | **—** |  | **—** |  |  |
| **Trauma-related factors** | | |  |  |  |  |  |  |  |
|  | Road user type (Ref: Driver) | |  |  |  |  |  |  |  |
|  |  | Cyclist | **†** |  | **—** |  | (0.017, 0.685) |  |  |
|  |  | Motorcyclist | **†** |  | **—** |  | (-0.151, 0.658) |  |  |
|  |  | Motor vehicle passenger | **†** |  | **—** |  | (-0.077, 0.585) |  |  |
|  |  | Pedestrian | **†** |  | **—** |  | (-0.682, 0.058) |  |  |
|  | ISS | | **†** |  | (-0.222, -0.054) |  | **—** |  |  |
|  | Injury pain (VAS) | | **†** |  | (-0.875, -0.429) |  | **—** |  |  |
|  | Neck injury (Ref: No) | |  |  |  |  |  |  |  |
|  |  | Yes | **†** |  | (-2.904, -0.540) |  | **—** |  |  |
|  | Spine/back injury (Ref: No) | |  |  |  |  |  |  |  |
|  |  | Yes | **†** |  | (-2.761, -0.347) |  | **—** |  |  |
|  | Lower extremity injury (Ref: No) | |  |  |  |  |  |  |  |
|  |  | Yes | **†** |  | (-2.379, -0.116) |  | **—** |  |  |
|  | Recovery expectations (Ref: Less than 1 month) | |  |  |  |  |  |  |  |
|  |  | More than 1 month | **†** |  | (-6.463, -2.976) |  | (0.147, 0.919) |  |  |
|  |  | No idea | **†** |  | (-5.552, -2.849) |  | (-0.103, 0.524) |  |  |
|  | **—**Not significant in the final model.  **†** Not entered in the first model.  LGCM: latent growth curve model; EQ-VAS: EQ-5D-5L visual analog scale; RT: road trauma; Ref: reference; SE: standard error; PHQ-15: Patient Health Questionnaire-15; PHQ-4: Patient Health Questionnaire-4; VAS: visual analog scale. | | | | | | | | |

| **S5 Table. Results of final piece-wise LGCM for assessing the concurrent effect of predictors on changes in EQ-5D-5L summary score in imputed data (*n* = 1071).** | | | | | | | | |
| --- | --- | --- | --- | --- | --- | --- | --- | --- |
|  | | | Intercept  (pre-injury) | | Slope 1  (phase 1 post-injury: worsening) | | Slope 2  (phase 2 post-injury: improving) | |
|  | | | Estimate (SE) | P-value | Estimate (SE) | P-value | Estimate (SE) | P-value |
| **Sociodemographic factors** | | |  |  |  |  |  |  |
|  | Sex (Ref: Male) | |  |  |  |  |  |  |
|  |  | Female | **—** | **—** | -0.017 (0.006) | 0.006 | **—** | **—** |
|  | Employment status (Ref: Employed) | |  |  |  |  |  |  |
|  |  | School | **—** | **—** | 0.019 (0.009) | 0.033 | **—** | **—** |
|  |  | Retired | **—** | **—** | 0.003 (0.008) | 0.662 | **—** | **—** |
|  |  | Other | **—** | **—** | 0.002 (0.013) | 0.901 | **—** | **—** |
|  | Education level (Ref: University) | |  |  |  |  |  |  |
|  |  | Less than high school | **—** | **—** | **—** | **—** | -0.003 (0.001) | 0.015 |
|  |  | High school or vocational | **—** | **—** | **—** | **—** | -0.007 (0.003) | 0.009 |
|  | Ethnicity (Ref: White) | |  |  |  |  |  |  |
|  |  | Asian | -0.001 (0.005) | 0.834 | **—** | **—** | **—** | **—** |
|  |  | Other | 0.009 (0.004) | 0.009 | **—** | **—** | **—** | **—** |
|  | Living situation (Ref: With others) | |  |  |  |  |  |  |
|  |  | Alone | -0.011 (0.005) | 0.020 | **—** | **—** | **—** | **—** |
|  | Years lived in Canada (Ref: >10 years) | |  |  |  |  |  |  |
|  |  | ≤10 years | 0.007 (0.003) | 0.020 | **—** | **—** | **—** | **—** |
| **Psychological and medical factors** | | |  |  |  |  |  |  |
|  | Somatic symptom severity (PHQ-15) | | -0.004 (0.001) | <0.001 |  |  | **—** | **—** |
|  | Pain catastrophizing | | -0.001 (0.0001) | 0.003 | **—** | **—** | **—** | **—** |
|  | Psychological distress (PHQ-4) | | -0.01 (0.002) | <0.001 | **—** | **—** | **—** | **—** |
|  | Pre-injury body complaints (Ref: No) | |  |  |  |  |  |  |
|  | Yes | | -0.034 (0.006) | 0.005 | 0.020 (0.008) | 0.003 | **—** | **—** |
|  | Pre-injury medication use (Ref: No) | |  |  |  |  |  |  |
|  | Yes | | -0.011 (0.004) | 0.033 | **—** | **—** | **—** | **—** |
| **Trauma-related factors** | | |  |  |  |  |  |  |
|  | ISS | | **†** | **†** | -0.003 (0.001) | <0.001 | 0.0001 (0.0001) | <0.001 |
|  | Injury pain (VAS) | | **†** | **†** | -0.010 (0.001) | <0.001 | **—** | **—** |
|  | Neck injury (Ref: No) | |  |  |  |  |  |  |
|  |  | Yes | **†** | **†** | -0.016 (0.006) | 0.011 | **—** | **—** |
|  | Spine/back injury (Ref: No) | |  |  |  |  |  |  |
|  |  | Yes | **†** | **†** | -0.019 (0.007) | 0.004 | **—** | **—** |
|  | Upper extremity injury (Ref: No) | |  |  |  |  |  |  |
|  |  | Yes | **†** | **†** | -0.015 (0.007) | 0.025 | 0.004 (0.001) | 0.012 |
|  | Lower extremity injury (Ref: No) | |  |  |  |  |  |  |
|  |  | Yes | **†** | **†** | -0.025 (0.007) | <0.001 | 0.003 (0.001) | 0.015 |
|  | Recovery expectations (Ref: Less than 1 month) | |  |  |  |  |  |  |
|  |  | More than 1 month | **†** | **†** | -0.043 (0.010) | <0.001 | 0.003 (0.002) | 0.067 |
|  |  | No idea | **†** | **†** | -0.036 (0.008) | <0.001 | 0.001 (0.001) | 0.407 |
|  | **—**Not significant in the final model.  **†** Not entered in the first model.  LGCM: latent growth curve model; EQ-5D-5L: European Quality of Life-5 Dimensions; RT: road trauma; Ref: reference; SE: standard error; PHQ-15: Patient Health Questionnaire-15; PHQ-4: Patient Health Questionnaire-4; ISS: injury severity score; VAS: visual analog scale. | | | | | | | |

| **S6 Table. Results of final piece-wise LGCM for assessing the concurrent effect of predictors on changes in EQ-VAS score in imputed data (*n* = 1067).** | | | | | | | | | |
| --- | --- | --- | --- | --- | --- | --- | --- | --- | --- |
|  | | | Intercept  (pre-injury) | | Slope 1  (phase 1 post-injury: worsening) | | Slope 2  (phase 2 post-injury: improving) | | |
|  | | | Estimate (SE) | P-value | Estimate (SE) | P-value | Estimate (SE) | P-value | |
| **Sociodemographic factors** | | |  |  |  |  |  |  | |
|  | Sex (Ref: Male) | |  |  |  |  |  |  |  |
|  |  | Female | 1.95 (0.60) | <0.001 | -1.69 (0.62) | 0.006 | **—** | **—** |  |
|  | Ethnicity (Ref: White) | |  |  |  |  |  |  |  |
|  |  | Asian | -0.12 (0.75) | 0.863 | **—** | **—** | **—** | **—** |  |
|  |  | Other | 2.15 (0.74) | <0.001 | **—** | **—** | **—** | **—** |  |
| **Psychological and medical factors** | | |  |  |  |  |  |  |  |
|  | Somatic symptom severity (PHQ-15) | | -1.14 (0.13) | <0.001 | 0.30 (0.12) | 0.006 | **—** | **—** |  |
|  | Pain catastrophizing | | -0.16 (0.04) | <0.001 | **—** | **—** | **—** | **—** |  |
|  | Pre-injury comorbidities number | | -1.43 (0.33) | <0.001 | 0.54 (0.19) | 0.016 | **—** | **—** |  |
|  | Pre-injury medication use (Ref: No) | |  |  |  |  |  |  |  |
|  |  | Yes | -2.45 (0.64) | <0.001 | **—** | **—** | **—** | **—** |  |
| **Trauma-related factors** | | |  |  |  |  |  |  | |
|  | Road user type (Ref: Driver) | |  |  |  |  |  |  |  |
|  |  | Cyclist | **†** | **†** | **—** | **—** | 0.33 (0.16) | 0.028 |  |
|  |  | Motorcyclist | **†** | **†** | **—** | **—** | 0.25 (0.20) | 0.253 |  |
|  |  | Motor vehicle passenger | **†** | **†** | **—** | **—** | 0.23 (0.15) | 0.178 |  |
|  |  | Pedestrian | **†** | **†** | **—** | **—** | -0.29 (0.21) | 0.137 |  |
|  | ISS | | **†** | **†** | -0.15 (0.05) | 0.002 | **—** | **—** |  |
|  | Injury pain (VAS) | | **†** | **†** | -0.65 (0.11) | <0.001 | **—** | **—** |  |
|  | Neck injury (Ref: No) | |  | **†** |  |  |  |  |  |
|  |  | Yes | **†** | **†** | -1.69 (0.64) | 0.010 | **—** | **—** |  |
|  | Spine/back injury (Ref: No) | |  | **†** |  |  |  |  |  |
|  |  | Yes | **†** | **†** | -1.45 (0.60) | 0.015 | **—** | **—** |  |
|  | Lower extremity injury (Ref: No) | |  | **†** |  |  |  |  |  |
|  |  | Yes | **†** | **†** | -1.16 (0.56) | 0.037 | **—** | **—** |  |
|  | Recovery expectations (Ref: Less than 1 month) | |  | **†** |  |  |  |  |  |
|  |  | More than 1 month | **†** | **†** | -4.71 (0.84) | <0.001 | 0.55 (0.18) | 0.003 |  |
|  |  | No idea | **†** | **†** | -4.18 (0.70) | <0.001 | 0.19 (0.17) | 0.197 |  |
|  | **—**Not significant in the final model.  **†** Not entered in the first model.  LGCM: latent growth curve model; EQ-VAS: EQ-5D-5L visual analog scale; RT: road trauma; Ref: reference; SE: standard error; PHQ-15: Patient Health Questionnaire-15; PHQ-4: Patient Health Questionnaire-4; VAS: visual analog scale. | | | | | | | |  |

**4. Software Code**

**R Code for Generalized Additive Mixed Model (GAMM)**

gamm_model <- gam(

index ~ s(time, bs = "tp", k = 5) +

s(Subject, bs = "re") + # Random intercept

s(time, Subject, bs = "fs"), # Random smooth for time per subject

data = data_clean,

method = "REML"

)

**Mplus Syntax for Conditional Piecewise Latent Growth Model (LGM)**

TITLE: Conditional Piecewise Latent Growth Model (LGM)

DATA: FILE = EQ-5D-5L.index.dat;

VARIABLE:

NAMES = subject I1-I5 x1-x35;

MISSING = ALL (999);

USEVAR = I1-I5 x2-x4 x6-x15 x19-x20 x27 x29-x35;

ANALYSIS: ESTIMATOR = MLR;

MODEL:

eta0 eta1 | I1@0 I2@2 I3@2 I4@2 I5@2;

eta0 eta2 | I1@0 I2@0 I3@2 I4@4 I5@10;

eta0 WITH eta1@0;

[ I2 ];

eta0 ON x2-x4 x12-x15 x19-x20;

eta1 ON x6-x9 x19 x27 x29-x35;

eta2 ON x10 x11 x30-x34;

OUTPUT: TECH1 TECH4;
